# The pro-apoptotic JNK scaffold POSH/SH3RF1 mediates *CHMP2B^Intron5^*-associated toxicity in animal models of frontotemporal dementia

**DOI:** 10.1093/hmg/ddy048

**Published:** 2018-02-08

**Authors:** Ryan J H West, Chris Ugbode, Fen-Biao Gao, Sean T Sweeney

**Affiliations:** 1 Department of Biology, University of York, York YO10 5DD, UK; 2 Department of Neurology, University of Massachusetts Medical School, Worcester, MA 01605, USA

## Abstract

Frontotemporal dementia (FTD) is one of the most prevalent forms of early-onset dementia. However, the pathological mechanisms driving neuronal atrophy in FTD remain poorly understood. Here we identify a conserved role for the novel pro-apoptotic protein plenty of SH3s (POSH)/SH3 domain containing ring finger 1 in mediating neuropathology in *Drosophila* and mammalian models of charged multivesicular body protein 2B (*CHMP2B^Intron5^*) associated FTD. Aberrant, AKT dependent, accumulation of POSH was observed throughout the nervous system of both *Drosophila* and mice expressing *CHMP2B^Intron5^*. Knockdown of POSH was shown to be neuroprotective and sufficient to alleviate aberrant neuronal morphology, behavioral deficits and premature-lethality in *Drosophila* models, as well as dendritic collapse and cell death in *CHMP2B^Intron5^*expressing rat primary neurons. POSH knockdown also ameliorated elevated markers of Jun N-terminal kinase and apoptotic cascades in both *Drosophila* and mammalian models. This study provides the first characterization of POSH as a potential component of an FTD neuropathology, identifying a novel apoptotic pathway with relevance to the FTD spectrum.

## Introduction

Frontotemporal dementia (FTD), a clinically, genetically and pathologically heterogeneous neurodegenerative disease, is a common form of early-onset dementia. FTD refers to a group of clinical syndromes associated with frontotemporal lobar degeneration (FTLD), a progressive degeneration of the frontal and temporal lobes of the brain. The principal syndromes associated with FTLD include behavioral variant FTD (bvFTD), progressive non-fluent aphasia, semantic dementia and FTD with motor neuron disease (FTD-MND). bvFTD is the most prevalent, accounting for ∼60% of all FTD cases. Perturbed regulation of apoptosis is a proposed mechanism underpinning neuronal death in FTD and has been observed in different FTD variants (1–7). A number of FTD loci are implicated in neuronal apoptosis [Valosin containing protein (VCP), TANK binding kinase 1 (TBK1), granulin precursor (GRN)], however, the cellular machinery driving the pro-apoptotic signal has yet to be determined (8,9).

Previously we established a *Drosophila* model of FTD associated with the bvFTD-disease causing mutation charged multivesicular body protein 2B (*CHMP2B^Intron5^*) (7,10,11). *CHMP2B^Intron5^*causes a C-terminal truncation of the CHMP2B protein and failure of CHMP2B to dissociate from the endosomal sorting complex required for transport III complex (12,13). Using this model we demonstrated *CHMP2B^Intron5^* perturbs normal endosomal and autophagic trafficking (7,10,11). Neuronal loss through phagocytic clearance of apoptotic neurons has been observed in *CHMP2B^Intron5^* models (14), however the mechanisms driving this process are not fully established. Previously we found the pro-apoptotic Jun N-terminal kinase (JNK) scaffold plenty of SH3’s/SH3 domain containing ring finger 1 (POSH/SH3RF1) is activated in *Rab8* mutants (7), dominant enhancers of *CHMP2B^Intron5^*. We now show POSH accumulates in the nervous system and mediates toxicity in *Drosophila* and mammalian models of *CHMP2B^Intron5^*.

POSH forms a scaffold for a multi-protein complex involved in JNK and NF-κB dependent apoptosis (15–17). This complex assembles following apoptotic stimuli and stabilizes through association with JNK components (18–22). This self-amplifying feedback loop leads to JNK-dependent apoptosis and cell death (22). POSH overexpression induces neuronal apoptosis while knockdown conveys neuroprotection against ischemia (16,23,24). AKT inhibits POSH dependent apoptosis by promoting disassembly of the pro-apoptotic POSH–JNK complex (18–20).

Using our *Drosophila* model of *CHMP2B^Intron5^* dominant screens were performed, identifying loci modifying *CHMP2B^Intron5^* toxicity (7,10,11). This identified the pro-survival gene AKT as a potent modifier of *CHMP2B^Intron5^* toxicity. Having shown accumulation of POSH to mediate neuronal dysfunction in *Rab8* mutants (7), another dominant modifier of CHM2B^Intron5^, this study looked to elucidate a role for POSH as a component of neuropathological and pro-apoptotic cascades in FTD. Using *Drosophila*, primary mammalian neurons and neuronal tissue from *CHMP2B^Intron5^* expressing mice we demonstrate POSH to be a conserved component of pathology in *CHMP2B^Intron5^ models.* We show POSH accumulates in neurons and drives unregulated synaptic growth, behavioral dysfunction and early-lethality in flies. POSH knockdown ameliorated dendritic collapse in primary mammalian neurons expressing CHMP2B^Intron5^. In keeping with the pro-apoptotic function of POSH we demonstrate expression of *CHMP2B^Intron5^* results in elevated JNK and apoptotic markers, both of which can be alleviated by POSH knockdown. This study implicates POSH as an important component of toxicity in *CHMP2B^Intron5^* FTD, defining a novel pathway potentially mediating toxicity and neuronal survival in FTD.

## Results

### A genetic screen in *Drosophila* identifies *AKT* as a dominant modifier of *CHMP2B^Intron5^* toxicity

**Figure 1.**
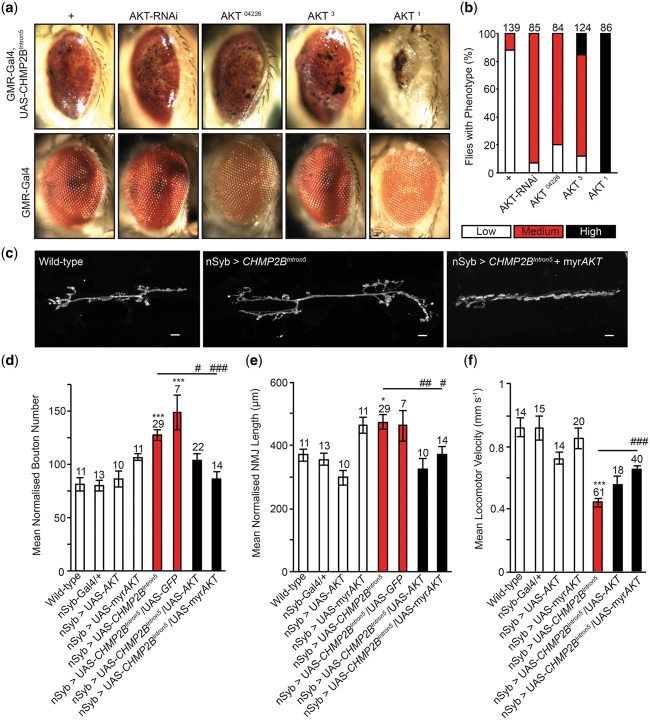
AKT is a dominant modifier of *CHMP2B^Intron5^*. (**A**) Representative images showing dominant enhancement of the *Drosophila* eye phenotype, associated with expression of the FTD disease-causing *CHMP2B^Intron5^* transgene in the *Drosophila* eye (GMR-Gal4), by AKT knockdown (RNAi), loss-of-function alleles (AKT^04226^ and AKT^3^) and the AKT kinase-dead allele (AKT^1^). (**B**) Quantification of the eye phenotype. (**C**–**E**) Co-expression of AKT or constitutively active myrAKT alleviates increased mean normalized synaptic bouton number (**D**) and NMJ length (E) at the *Drosophila* third instar larval neuromuscular junction (hemi-segment A3, muscle 6/7) in animals pan-neuronally (nSyb-Gal4) expressing the *CHMP2B^Intron5^* transgene. NMJ’s were analyzed across a minimum of five animals. One-way ANOVA with Dunnett’s post hoc comparison to wild-type controls (*P < 0.05, ***P < 0.001) and Tukey between groups comparison (^#^*P* < 0.05, ^##^*P* < 0.01 and ^###^*P* < 0.001). (**F**) Reduced locomotor velocity observed in larvae pan-neuronally (nSyb-Gal4) expressing *CHMP2B^Intron5^* is ameliorated by co-expression of AKT or myrAKT. One-way ANOVA with Dunnett’s post hoc comparison to wild-type controls (***P < 0.001) and Tukey between groups comparison (^###^*P* < 0.001). Scale bars = 10 μm. Error bars represent SEM, sample size is reported above each bar.

Expression of the disease-causing *CHMP2B^Intron5^* mutant transgene in the *Drosophila* eye elicits a perturbed eye phenotype, described previously (7,10,11). This phenotype allowed us to screen ∼ 80% of the *Drosophila* genome for dominant modifiers of *CHMP2B^Intron5^* toxicity (7,10,11). Here we identify *AKT* as a dominant enhancer of toxicity, with *AKT* loss-of-function alleles *AKT^04226^* and *AKT^3^* and AKT knockdown, via RNA interference (RNAi), potentiating the *CHMP2B^Intron5^* eye phenotype (Fig. 1A and B). The kinase-dead *AKT* allele *AKT^1^* showed the most significant enhancement of the eye phenotype (Fig. 1A and B;^25^).

We previously demonstrated pan-neuronal expression of *CHMP2B^Intron5^* results in unregulated synaptic overgrowth at the *Drosophila* larval neuromuscular junction (NMJ) (7)*.* We therefore employed this model to establish whether expression of AKT could alleviate neuronal aberrations in larvae pan-neuronally [neuronal synaptobrevin (*nSyb*)-Gal4] expressing *CHMP2B^Intron5^*. Expression of AKT or constitutively active myristoylated AKT (myrAKT) was sufficient to alleviate synaptic overgrowth, reducing both elevated synaptic bouton number and NMJ length (Fig. 1C–E). Co-expression of upstream activator sequence (UAS)-mCD8-GFP with *CHMP2B^Intron5^* showed no variance to *CHMP2B^Intron5^* expression alone, indicating rescues were not the result of titrating Gal4. To provide a functional, behavioral readout of NMJ activity larval locomotor assays were employed. *CHMP2B^Intron5^* expressing larvae displayed significantly reduced larval crawling velocity, compared with wild-types or controls (Fig. 1F). Co-expression of AKT or myrAKT with *CHMP2B^Intron5^* was sufficient to partially rescue locomotor deficits, with the myrAKT providing the most significant rescue (Fig. 1F). 

### AKT mediates aggregation of the pro-apoptotic JNK scaffold POSH

**Figure 2.**
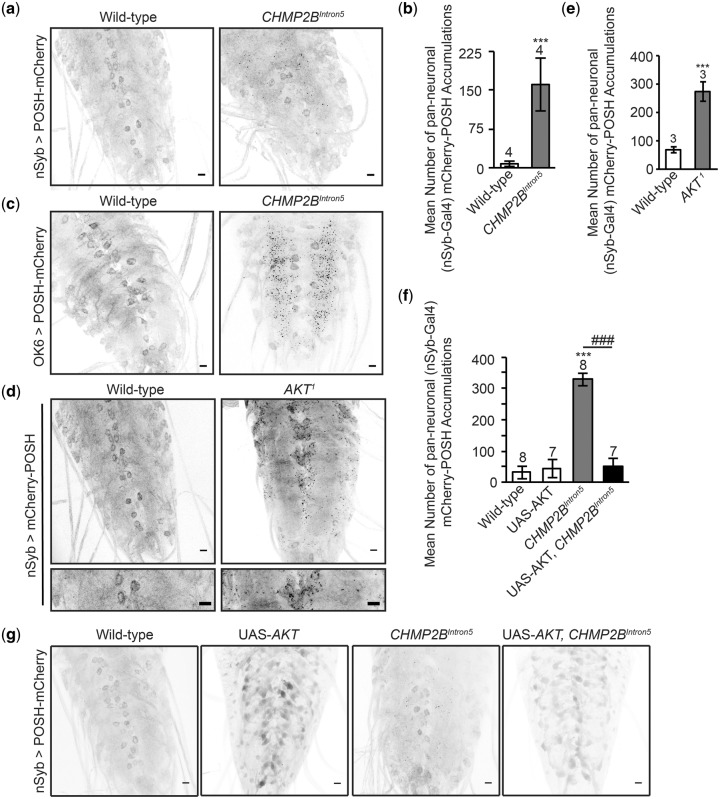
AKT, a negative regulator of POSH, alleviates aberrant accumulation of POSH in the nervous system of *Drosophila* expressing the disease-causing *CHMP2B^Intron5^* transgene. (**A** and **B**) Pan-neuronal (nSyb-Gal4) expression of the *CHMP2B^Intron5^* mutant transgene results in a significant accumulation of mCherry-POSH positive puncta throughout the ventral nerve cord of third instar *Drosophila* larvae (Student’s *t*-test, ***P < 0.001). Aberrant accumulation of POSH puncta was also observed in the ventral nerve cord of flies expressing *CHMP2B^Intron5^* within motor neurons (OK6-Gal4) (**C**) and in AKT kinase-dead (AKT^1^) larvae (Student’s *t*-test, ***P < 0.001) (**D** and **E**). (**F** and **G**) Co-expression of AKT significantly reduces the number of POSH positive accumulations observed in the ventral nerve cord of larvae pan-neuronally (nSyb-Gal4) expressing the *CHMP2B^Intron5^* mutant transgene. One-way ANOVA with Dunnett’s post hoc comparison to wild-type controls (***P < 0.001) and Tukey between groups comparison (^###^*P* < 0.001). Scale bars = 10 μm. Error bars represent SEM, sample size is reported above each bar.

AKT exhibits anti-apoptotic activity in neurons through direct interaction and phosphorylation of POSH (18). Previously we demonstrated POSH accumulation in the nervous system of larvae mutant for *Rab8*, a dominant modifier of *CHMP2B^Intron5^* (7). Rab8 also interacts with TBK1, Optineurin and C9orf72, known loci causing FTD and MND (26–28). We therefore asked whether POSH accumulation was also observed in the nervous system of larvae expressing *CHMP2B^Intron5^*, in a manner phenocopying *Rab8* mutants. Pan-neuronal expression of *CHMP2B^Intron5^* resulted in aberrant accumulation of POSH in distinct puncta throughout the larval ventral nerve cord (VNC) (Fig. 2A). Puncta were most frequently observed within the neuropil, mimicking *Rab8* mutants, which show POSH accumulations throughout their axons. Quantification revealed a significant increase in POSH accumulations in the VNC of *CHMP2B^Intron5^* expressing animals, compared with wild-type (Fig. 2B). Having observed pan-neuronal expression of *CHMP2B^Intron5^* perturbs synaptic structure and function at the *Drosophila* larval NMJ (Fig. 1C–F) we also asked whether *CHMP2B^Intron5^*expression within motor neurons, using the OK6-Gal4 Driver, induced aberrant accumulation of POSH. Motor neuronal expression of *CHMP2B^Intron5^*was sufficient to induce aberrant accumulation of POSH puncta throughout the neuropil region of the VNC (Fig. 2C).

POSH is a substrate of AKT *in vitro* and in cell culture, with phosphorylation of POSH by AKT negatively regulating assembly of the pro-apoptotic POSH complex (18). We therefore looked to ascertain whether this interaction occurred *in vivo* in the *Drosophila* nervous system and whether inhibition of AKT kinase function affected POSH accumulation. The *Drosophila AKT^1^* mutant allele is characterized by a point mutation leading to a single amino-acid change (F327I) within a highly conserved region of the kinase catalytic core domain (25). This mutation results in a complete loss of kinase activity and is considered a kinase-dead allele. POSH was shown to accumulate throughout the larval VNC of *AKT^1^* kinase-dead flies in a manner phenocopying *CHMP2B^Intron5^* (Fig. 2D). No significant accumulation of POSH was observed in wild-type animals. Immunoblotting using the anti-Phospho-(Ser/Thr) Akt Substrate Antibody, which preferentially recognizes peptides phosphorylated by AKT, following immunoprecipitation of POSH from *Drosophila* lysates, confirmed POSH to be a direct substrate of AKT *in vivo* (18; Supplementary Material, Fig. S1A). 

Having observed POSH accumulations in the nervous system of *AKT^1^* kinase dead flies (Fig. 2D) and demonstrated expression of AKT to be sufficient to rescue synaptic overgrowth at the *Drosophila* larval NMJ in *CHMP2B^Intron5^* expressing animals (Fig. 1C–E), we looked to ascertain whether AKT expression could alleviate aberrant accumulation of POSH in the nervous system of *CHMP2B^Intron5^* expressing flies. Expression of AKT was sufficient to reduce the number of distinct POSH puncta observed within the VNC and resulted in a re-distribution of POSH to a more diffuse cytosolic localization (Fig. 2E and F). This was supported by our observation that overexpression of myrAKT was able to rescue reduced phosphorylation of POSH observed in lysates extracted from the VNC of *CHMP2B^Intron5^* expressing larvae (Supplementary Material, Fig. S1B).

### Knockdown of POSH alleviates *CHMP2B^Intron5^* toxicity in *Drosophila*

**Figure 3.**
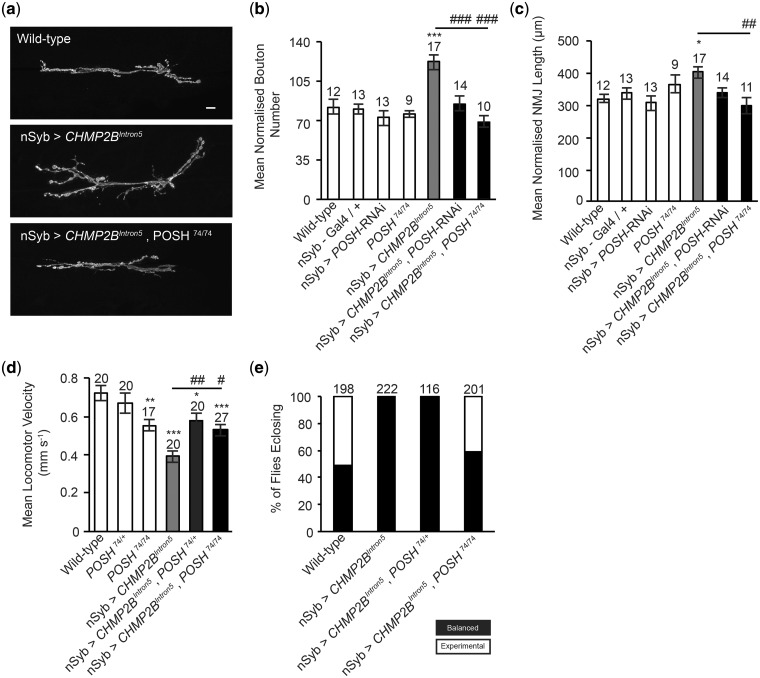
POSH knockdown alleviates *CHMP2B^Intron5^* phenotypes in *Drosophila.* (**A–C**) Knockdown of POSH via POSH-RNAi or using the strong hypomorphic allele POSH^74^ rescues the increased mean normalized synaptic bouton number (**B**) and mean normalized NMJ length (**C**) observed in larvae pan-neuornally (nSyb-Gal4) expressing the *CHMP2B^Intron5^* mutant transgene (muscle 6/7, hemi-segment A3). NMJ analysis was performed across a minimum of five individual animals. One-way ANOVA with Dunnett’s post hoc comparison to wild-type controls (*P < 0.05, ***P < 0.001) and Tukey between groups comparison (^##^*P* < 0.01, ^###^*P* < 0.001). Scale bars = 10 μm. (**D**) Knockdown of POSH ameliorates impaired larval locomotion in larvae pan-neuronally (nSyb-Gal4) expressing the *CHMP2B^Intron5^* mutant transgene. One-way ANOVA with Dunnett’s post hoc comparison to wild-type controls (*P < 0.05, **P < 0.01, ***P < 0.001) and Tukey between groups comparison (^#^*P* < 0.05, ^##^*P* < 0.01). (**E**) Pan-neuronal expression of the *CHMP2B^Intron5^* mutant transgene results in a 100% pharate (pupal) lethal phenotype which can be ameliorated by knockdown of POSH using a homozygous POSH^74^ hypomorphic allele (POSH^74/74^). Error bars represent SEM, sample size is reported above each bar.

Having confirmed that POSH is a direct substrate of AKT and shown that AKT expression is sufficient to alleviate synaptic perturbations and aberrant localization of POSH in *CHMP2B^Intron5^* expressing larvae, we asked whether POSH played a critical role in driving pathological pathways in *CHMP2B^Intron5^* expressing animals. Using the *Drosophila* larval NMJ as a model synapse we demonstrate knockdown of POSH via RNAi or using the previously characterized hypomorphic allele *POSH^74^* (7,29) was sufficient to alleviate all aspects of synaptic overgrowth observed in *CHMP2B^Intron5^* expressing larvae, including increased synaptic bouton number and synapse length (Fig. 3A–C). Knockdown of POSH also alleviated impaired larval crawling in *CHMP2B^Intron5^* expressing larvae (Fig. 3D).

In addition to neuroanatomical perturbations at the *Drosophila* larval NMJ pan-neuronal expression of *CHMP2B^Intron5^* results in a 100% pharate (adult pupal) lethal phenotype. In order to ascertain whether inhibition of POSH could reduce the penetrance of this lethal phase, survival assays were performed. Experimental crosses were designed to give a 50:50 ratio of progeny either pan-neuronally expressing *CHMP2B^Intron5^* or siblings not expressing *CHMP2B^Intron5^* but carrying an identifiable marker (*CyO*, balancer chromosome with curly wings). The effect of reducing POSH expression, using both heterozygous and homozygous POSH mutants, on survival was then assessed in these backgrounds by scoring the number of flies with normal or curly wings eclosing as adults. A wild-type cross giving a 50:50 ratio of straight: curly winged flies was used as a baseline control. Pan-neuronal expression of *CHMP2B^Intron5^* resulted in a 100% lethal phenotype, with all eclosing offspring carrying the *CyO* balancer (Fig. 3E). In contrast, homozygous *POSH (POSH ^74/74^)* mutants pan-neuronally expressing *CHMP2B^Intron5^* showed reduced lethality, with 40% of eclosing flies expressing the *CHMP2B^Intron5^*transgene (Fig. 3E).

### Neuronal accumulation of POSH mediating toxicity is conserved in mammalian models of *CHMP2B^Intron5^* FTD

**Figure 4.**
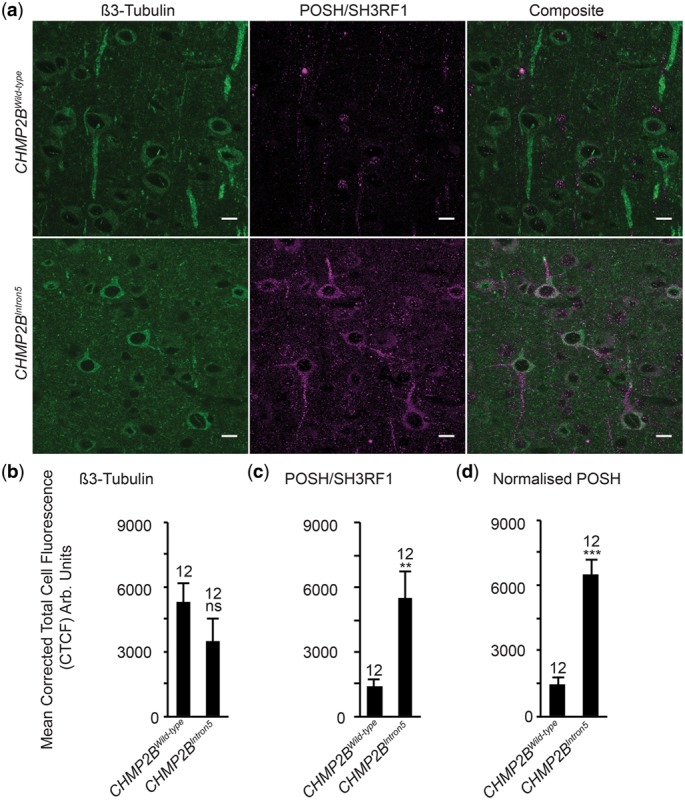
Aberrant neuronal accumulation of POSH is conserved in *CHMP2B^Intron5^* mice (**A**–**D)** representative images and quantification, via corrected total cell fluorescence (CTCF), of POSH within β3 tubulin positive neurons in the frontal cortex of 12-month-old mice expressing either *CHMP2B^Intron5^* or CHMP2B^Wiltype^ under the control of the Camk2a promoter. (D) Relative fluorescence intensity of POSH (**C**) was normalized against relative fluorescence intensity of β3 tubulin (B). Student’s *t*-test **P < 0.01, ***P < 0.001; *n* = 12, *N* = 3. Scale bars = 10 μm. Error bars represent SEM.

Having observed aberrant accumulation of POSH within the nervous system of flies expressing *CHMP2B^Intron5^* we asked whether this phenotype was conserved in mammalian CHMP2B^Intron5^ models. We therefore employed our previously established *CHMP2B^Intron5^* mouse model (30). POSH accumulation was observed in β3-tubulin positive neurons within the frontal cortex of 12-month-old mice expressing *CHMP2B^Intron5^* but not aged matched *CHMP2B^Wild-type^* expressing controls (Fig. 4). Accumulations were specific to the cell body and neuronal processes. Quantification of the relative fluorescence abundance of POSH within β3-tubulin positive neurons showed a significant increase in *CHMP2B^Intron5^* expressing mice, compared with *CHMP2B^Wild-type^*controls. β3-Tubulin expression showed no significant variance to wild-type.

**Figure 5.**
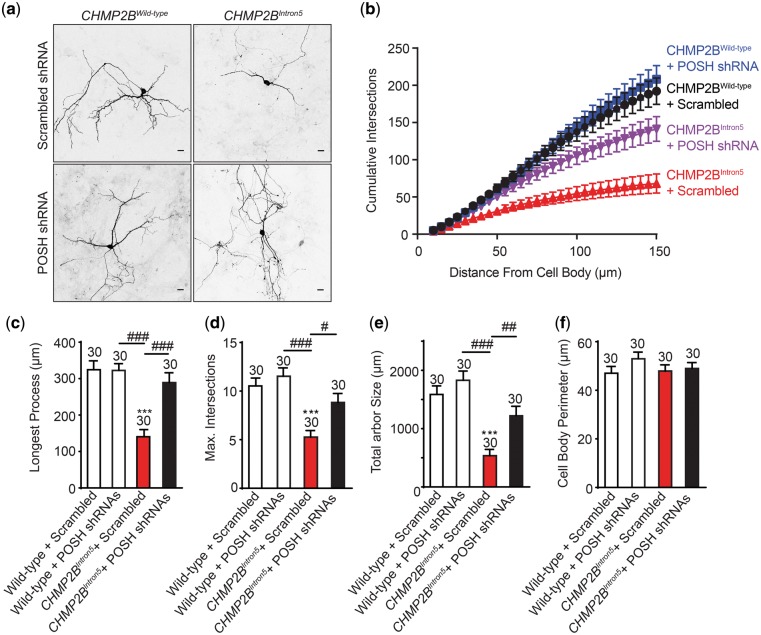
POSH knockdown alleviates *CHMP2B^Intron5^* dependent dendritic collapse in mammalian neurons. (**A**) Representative micrographs of mature neurons expressing FLAG-tagged *CHMP2B^Wild-type^* or *CHMP2B^Intron5^* in the presence of either pSIREN-RetroQ-DsRed-Scrambled or POSH 1 + 2 shRNA. Scale bars = 20μm. *CHMP2B^Intron5^* shows significant reduction in cumulative branch number (compared with *CHMP2B^Wild-type^* + Scrambled (*P* < 0.001) and *CHMP2B^Intron5^*+ POSH shRNA’s (*P* < 0.001)) (**B**), longest process length (**C**) maximum number of dendritic branches (**D**) and total arbor size (**E**) without affecting cell body size (**F**). (C–F) One-way ANOVA with Dunnett’s post hoc comparison to wild-type controls (***P < 0.001) and Tukey between groups comparison (^#^*P* < 0.05, ^##^*P* < 0.01 and ^##^*P* < 0.001), *n* = 30 cells across three biological replicates. Error bars represent SEM, sample size is reported above each bar.

Previously we demonstrated that mammalian neurons transfected with *CHMP2B^Intron5^* show dendritic collapse prior to death (13). Similar phenotypes are observed via knockdown of *CHMP2B* (31). In order to ascertain whether POSH plays a functional role in *CHMP2B^Intron5^* toxicity in mammalian neurons, we asked whether knockdown of POSH via short hairpin RNA (shRNA) could alleviate dendritic collapse and convey neuroprotection (Fig. 5). Neurons transfected with *CHMP2B^Intron5^* showed a significant dendritic collapse phenotype compared with those transfected with wild-type *CHMP2B*. Sholl analysis revealed *CHMP2B^Intron5^* transfected neurons show reduced complexity of the dendritic arbor, with a significant decrease in the number of cumulative intersections observed compared with neurons transfected CHMP2B^Wild-type^ (Fig. 5A and B). *CHMP2B^Intron5^* transfected neurons also showed a significant reduction in the length of the longest neuronal process (Fig. 5A and C, 56% reduction), the maximum number of intersections at any given distance from the cell body (Fig. 5A and D, 50% reduction) and the total arbor size (Fig. 5A and E, 66% reduction). The total perimeter of the cell body showed no variance between neurons transfected with either *CHMP2B^Intron5^* or *CHMP2B^Wild-type^*. Co-transfection of *POSH* shRNA’s was sufficient to alleviate all aspects of perturbed dendritic morphology associated with expression of *CHMP2B^Intron5^* (Fig. 5).

### Knockdown of POSH alleviates elevated apoptotic Cascades in *CHMP2B^Intron5^* models

Previous studies have identified POSH as a pro-apoptotic JNK scaffold (15–17). Overexpression of POSH has been shown to induce neuronal apoptosis while knockdown conveys neuroprotection against ischemia (16,23,24). Having observed neuroprotection via knockdown of POSH in both *Drosophila* and mammalian models of *CHMP2B^Intron5^* FTD, we asked whether expression of *CHMP2B^Intron5^* resulted in elevated JNK and apoptotic activity in these models.

**Figure 6.**
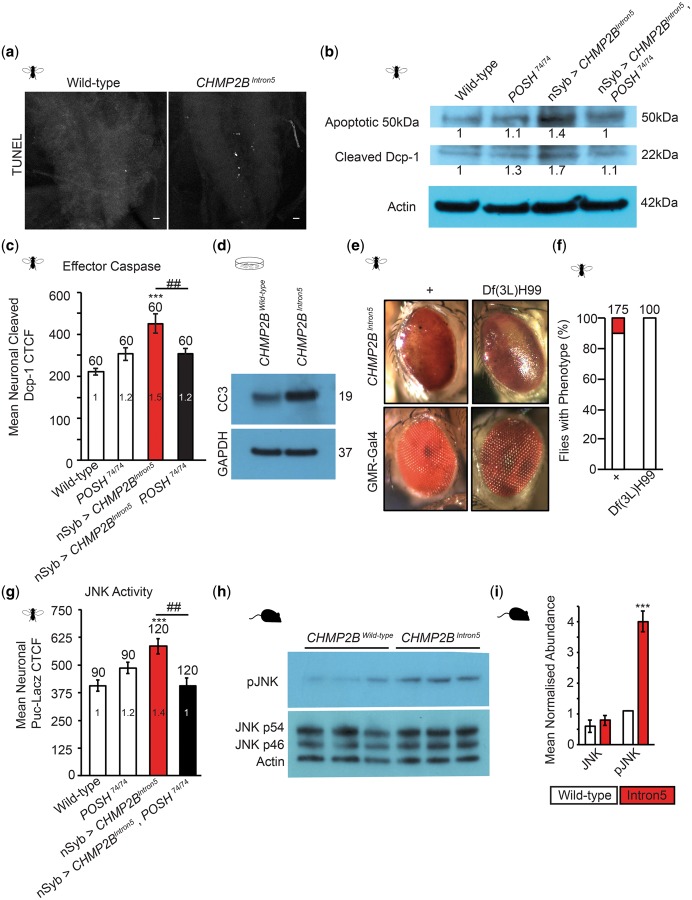
POSH knockdown alleviates aberrant apoptotic and JNK Activity in *Drosophila* and mammalian *CHMP2B^Intron5^* models. Pan-neuronal (nSyb-Gal4) expression of *CHMP2B^Intron5^* leads to an increase in apoptotic markers in the *Drosophila* third instar larval nervous system, including an increase in TUNEL (**A**) and cleaved Dcp-1, the *Drosophila* effector caspase, in the larval ventral nerve cord (**B** and **C**). Neuronal expression of *CHMP2B^Intron5^* also resulted in an increase in the apoptosis related 50 kDa band recognized by anti-Dcp-1 via immunoblotting (B). CTCF, corrected total cell fluorescence. Knockdown of POSH using the hypomorphic POSH^74^ allele ameliorates elevated cleaved Dcp-1 in animals expressing *CHMP2B^Intron5^* (B–C). One-way ANOVA with Dunnett’s post hoc comparison to wild-type controls (***P < 0.001) and Tukey between groups comparison (^##^*P* < 0.01). (**D**) Immunoblot showing cleaved-caspase 3 (CC3) in mammalian GPNT cells transfected with *CHMP2B^Wild-type^* and *CHMP2B^Intron5^*. (**E** and **F)** The deficiency locus Df(3L)H99, which ablates three critical apoptotic loci, as a heterozygote reduces the *CHMP2B^Intron5^* eye phenotype (GMR-Gal4). *Drosophila* and mouse models expressing *CHMP2B^Intron5^* show elevated levels of JNK activity within the nervous system. Elevated JNK activity, determined using the transcriptional reporter puckered-lacZ, in the ventral nerve cord of *Drosophila* third instar larvae pan-neuronally expressing *CHMP2B^Intron5^* (nSyb-Gal4) can be alleviated by POSH knockdown using the hypomorphic POSH^74^ allele (**G**). pJNK levels in cortical lysates extracted from 12-month-old mice expressing either *CHMP2B^Intron5^* or *CHMP2B^Wiltype^* under the control of the Camk2a promoter (**H**) and quantified relative to the actin loading control (**I**, *N* = 3; Student’s *t*-test, ***P <0.001). Error bars represent SEM, sample size is reported above each bar.

Pan-neuronal expression of *CHMP2B^Intron5^* resulted in an increase in terminal deoxynucleotidyl transferase (TdT)-mediated dUTP nick-end labeling (TUNEL) staining observed in the *Drosophila* larval VNC (Fig. 6A), coupled with a significant increase in the expression of cleaved Death Caspase 1 (Dcp-1) (Fig. 6B and C), the activated *Drosophila* effector caspase (32). Immunoblotting revealed a 1.7-fold increase in cleaved Dcp-1 in the larval VNC, compared with wild-type (Fig. 6B) while immunofluorescence quantification of cleaved Dcp-1 specifically within elav positive neurons in the VNC revealed a 1.5-fold increase (Fig. 6C). Reduction of POSH abundance in flies expressing *CHMP2B^Intron5^* using the hypomorphic POSH^74^ allele was sufficient to rescue cleaved Dcp-1 levels back to wild-type levels (Fig. 6B and C). Cleaved caspase 3 was also shown to be elevated in rat endothelial cells transfected with *CHMP2B^Intron5^*, but not *CHMP2B^Wild-type^*, supporting observations made in *Drosophila* (Fig. 6D). Inhibition of apoptosis using the deficiency allele Df(3L)H99, which has previously been shown to completely block apoptosis by deleting three essential pro-apoptotic genes (33), acted as a heterozygote to partially alleviate the *CHMP2B^Intron5^* eye phenotype, reducing the severity of the black spot phenotype observed (Fig. 6E and F).

Having observed increased apoptotic activity in both *Drosophila* and mammalian models of *CHMP2B^Intron5^* FTD, and taking into account the role of POSH as a pro-apoptotic JNK scaffold, we asked whether these models showed elevated JNK signaling and whether this could be alleviated by inhibition of POSH. Puckered (puc), a negative regulator of JNK transcriptionally activated by the JNK signaling pathway, has previously been shown to provide a reliable transcriptional readout of JNK activity (34,35). Using a *puc*-lacZ reporter (34) we demonstrate a significant increase in JNK activity within the nervous system of *CHMP2B^Intron5^* expressing larvae (Fig. 6G), supporting our previous observations (7). Inhibition of POSH was sufficient to alleviate elevated levels of *puc*. A significant increase in phospho-JNK was also observed in lysates extracted from the cortex of 12-month-old *CHMP2B^Intron5^* expressing mice, compared with aged matched CHMP2B^Wild-type^ expressing controls (Fig. 6H and I).

## Discussion

As part of an ongoing, functional screen to identify signaling mechanisms driving pathology in FTD we identified AKT as a potent modifier of *CHMP2B^Intron5^* toxicity. Expression of *CHMP2B^Intron5^* lead to aberrant accumulation of POSH, which is known to be negatively regulated by AKT (18,20), in the nervous system of both *Drosophila* and mammalian models. Having previously implicated POSH as a potential regulator of neuronal dysfunction (7) this study now defines, for the first time, the function of this novel regulator of apoptosis in an FTD model.

### AKT in *CHMP2B^Intron5^*-associated FTD

AKT has been implicated in the regulation of neuronal growth and survival as well as conveying neuroprotection in response to neuronal insults (36–39). It has been identified as a potential therapeutic target for neuroprotective compounds in response to ischemia and neurotoxic apoptosis (40). Perturbations to AKT function have been implicated in a number of neurodegenerative disorders including Alzheimer’s disease and FTD (41–44). It has also been show to directly interact with a number of proteins associated with FTD disease causing loci, including TBK1 and VCP (45–47). In this study, we identified the single *Drosophila* isoform of AKT, to be a potent modifier of *CHMP2B^Intron5^* toxicity. *AKT* loss-of-function mutants were shown to significantly enhance the eye phenotype associated with *CHMP2B^Intron5^* expression, revealing heterozygous mutations of AKT to be dominant enhancers of *CHMP2B^Intron5^* toxicity. The most significant enhancement to the eye phenotype was shown by the *AKT^1^* allele, an endogenous kinase-dead allele (25). This observation suggests an important functional role for AKT kinase activity in preventing neurotoxicity associated with the *CHMP2B^Intron5^* mutation. This is supported by the ability of both AKT and the constitutively active myristoylated AKT to rescue aberrant synaptic growth at the *Drosophila* larval NMJ, as well as impaired locomotor velocity. We also substantiate previous findings that POSH is a direct substrate of AKT and show that POSH aberrantly accumulates in the central nervous system of AKT kinase-dead flies. Despite this we did not observe changes in either AKT or phospho-AKT levels in *CHMP2B^Intron5^* models (data not shown). This suggests POSH accumulation in *CHMP2B^Intron5^* does not occur as a result of perturbed pro-survival AKT function, but more likely in response to endogenous pro-apoptotic stimuli promoting assembly of the active POSH complex. Activation of the pro-survival AKT pathway, however, is sufficient to alleviate POSH mediated toxicity, most likely through its known role as a negative regulator of POSH. This may therefore represent a pathway for therapeutic intervention. Similarly, disruption to AKT kinase function may potentiate disease severity by reducing negative regulation of the POSH signaling complex.

Additionally, we have demonstrated that reducing *POSH* expression alleviates synaptic overgrowth both in *CHMP2B^Intron5^* expressing flies and, previously, in *Rab8* mutants (7). Collectively these data reveal POSH as a novel candidate potentially acting downstream of AKT to modulate synaptic structural homeostasis. Given the known function of POSH as a JNK scaffold and the well-established role of the JNK-activator protein 1 pathway in regulating synaptic outgrowth at the *Drosophila* NMJ, POSH represents a promising candidate linking AKT to JNK-dependent regulation of NMJ morphology in an antagonistic manner. Given the conservation of AKT and JNK signaling pathways in neuronal growth and plasticity across species, observations made at the *Drosophila* larval NMJ may be directly translatable to a mammalian system and in the regulation of neuronal homeostasis in neuropathology (48,49).

### Modulation of POSH by AKT

Scaffolding proteins are post-translationally modified to modulate their activation state. AKT is as a negative regulator of the pro-apoptotic POSH-JNK signaling complex in the mammalian nervous system and in cell culture (18,20). Negative regulation of this complex occurs through phosphorylation of POSH and its interacting partners (18–20). Direct phosphorylation of POSH by AKT, however, has previously only been shown *in vitro* or in cell culture (18). Here, we provide evidence that POSH is an AKT substrate in the *Drosophila* nervous system and that inhibition of AKT kinase function leads to aberrant POSH accumulation. The observation that AKT expression alleviates POSH accumulation and toxicity in the nervous system of *CHMP2B^Intron5^* expressing flies also provides context for this pathway in FTD. Identification of this pathway in the regulation of neuronal growth and function in FTD model identifies the POSH signaling complex as a novel target mediating neuronal dysfunction and neurodegeneration in FTD.

### Inhibition of POSH is neuroprotective in *Drosophila* and mammalian models of *CHMP2B^Intron5^* FTD

POSH has been implicated in the development and maintenance of the nervous system, including neuronal migration and axon outgrowth (50–52). POSH knockdown conveys neuroprotection in response to neuronal insults, suggesting an important role for POSH in the regulation and survival of neurons (16,24). For example, knockdown of POSH is neuroprotective in response to cerebral ischemia and growth factor withdrawal (16,24). Conversely, overexpression of POSH induces caspase-dependent cell death (53). AKT is also implicated in neuroprotection in response to ischemia and growth factor withdrawal, supporting a conserved mechanism in which modulation of POSH by AKT promotes neuroprotection in response to neuronal insults (36–39). Here, we provide evidence that POSH knockdown is sufficient to alleviate neuronal perturbations in both *Drosophila* and mammalian models of FTD associated with the disease-causing *CHMP2B^Intron5^* mutation. POSH knockdown in *Drosophila* pan-neuronally expressing *CHMP2B^Intron5^* completely alleviated unregulated neuronal growth at the larval NMJ. Importantly POSH knockdown had no effect upon neuroanatomy in wild-type larvae suggesting a role for POSH in pathological neuronal dysfunction, rather than as a mediator of neuronal growth. POSH knockdown was also sufficient to ameliorate perturbed larval crawling and early lethality observed in *CHMP2B^Intron5^* flies, suggesting a pathological role for POSH leading to premature lethality in *Drosophila*. The observation that POSH knockdown ameliorated all aspects of the dendritic collapse phenotype observed in primary neurons transfected with *CHMPB^Intron5^* provides functional evidence for a conserved role of POSH in the transduction of neurotoxic pathways in both *Drosophila* and mammalian models of disease.

### POSH as a pro-apoptotic JNK scaffold in FTD

Premature apoptosis has been observed as an early event occurring in different FTD variants (1) and a number of FTD causing loci are implicated in neuronal apoptosis (VCP, TBK1, GRN) (8,9). Activation of microglia has also been shown to promote clearance of apoptotic neurons observed in the brains of 18-month-old *CHMP2B^Intron5^* mice, but not aged matched *CHMP2B^Wild-type^* or non-transgenic controls, indicating aberrant neuronal apoptosis may be driving cell-loss in *CHMP2B^Intron5^*-associated FTD (14). Mutations in *CHMP2B* have also been suggested to pre-dispose neurons to apoptosis (54). However, our understanding of whether apoptosis is driving cell death in FTD and the molecular machinery regulating this process remains poorly understood. Our observation that the pro-apoptotic JNK scaffold POSH aberrantly accumulates in both *Drosophila* and mammalian models of *CHMP2B^Intron5^* FTD and that POSH knockdown alleviates aberrant neuronal phenotypes identifies it as a potentially novel pro-apoptotic factor in FTD pathology.

POSH was initially identified in the regulation of JNK and NF-κB dependent apoptosis (15). POSH overexpression promotes caspase-dependent cell death, while knockdown promotes neuroprotection following neuronal insult (16,23,24). Ablation of SH3 domain containing ring finger 2 (SH3RF2), a negative regulator of POSH, leads to enhanced caspase-8 activity (55). Conversely expression of SH3RF2 prevents apoptosis and promotes neuronal cell survival through inhibition of POSH (21,55). The pro-apoptotic function of Nix/BNIP3L has also been shown to be dependent upon interaction with POSH (56). However, to date, POSH remains poorly studied and its role in neurodegenerative diseases remains unknown. This study is the first, to our knowledge, providing a functional context for POSH in a neurodegenerative disorder. We provide evidence that inhibition of POSH alleviates elevated caspase activity in both *Drosophila* and mammalian *CHMP2B^Intron5^*-induced FTD models. We also reveal inhibition of apoptosis using the *Df(3L)H99* deficiency locus, which ablates three essential apoptotic genes and reduces *CHMP2B^Intron5^* toxicity as a heterozygote. However, it is important to note that the *Df(3L)H99* allele does not completely alleviate the eye phenotype, suggesting a potential role for alternative pathways in *CHMP2B^Intron5^* toxicity. These may include non-canonical cell death pathways as well as autophagic pathways, which are known to be perturbed in *CHMP2B^Intron5^* models. Interestingly, the *Drosophila* effector caspase Dcp-1 has also been implicated in autophagic flux (57). This may represent a broader link to other mechanisms of cell death and autophagic disruption in FTD.

### Implications for FTD

In this study, we provide evidence for a functional, novel, role for the pro-apoptotic JNK scaffold POSH in mediating neuropathology in *Drosophila* and mammalian models of FTD associated with the disease-causing mutation *CHMP2B^Intron5^*. Aberrant apoptosis has been implicated as a potential mechanism driving neuronal cell death and gliosis in a number of FTD variants. The observation that POSH is perturbed in *CHMP2B^Intron5^*models therefore raises the question of whether this novel apoptotic-regulator has a functional role in other variants of the disease, or even more broadly in neurodegenerative diseases. Future investigation into the role of POSH in FTD and other neurodegenerative diseases, as well as whether aberrant POSH accumulation is conserved in patients, will be critical to elucidate the role of POSH in neurodegeneration. Further investigation into novel interacting partners of POSH in both healthy and diseased neurons may also help to delineate mechanisms regulating POSH and its downstream effects on neurodegeneration.

These observations provide the first characterization of POSH as a potential component of neuropathological cascades in FTD. It also reveals POSH as a novel target for further investigation and potential therapeutic intervention. Aberrant accumulation of POSH may also represent a biomarker of the disease though further investigation will be required to determine this.

## Materials and Methods

### Drosophila

#### Stocks and husbandry

*Drosophila* were raised on standard cornmeal–yeast–sucrose medium at 25°C on a 12 h light:dark cycle. CHMP2B^Intron5^ flies were described previously (7,10). All other stocks were obtained from the following sources: POSH^74^ (Toshiro Aigaki, Tokyo Metropolitan University, Japan) (29), AKT^1^ (Clive Wilson, University of Oxford, UK) (25), OK6-Gal4 (Cahir O’Kane, University of Cambridge, UK), UAS-myrAKT, UAS-mCD8-GFP, AKT^04226^, AKT^3^, UAS-AKT-RNAi (BL #33615), UAS-mCherry-POSH, UAS-POSH-RNAi (BL #64569), Df(3L)H99, Puc-LacZ, glass multimer reporter (GMR)-Gal4, nSyb-Gal4, Canton S, w^1118^ (Bloomington Stock Center). UAS-AKT (FlyORF, Zurich, Switzerland). All wild-types were an outcross of Canton S to w^1118^.

Genetic interaction experiments and quantification of the *CHMP2B^Intron5^* eye phenotype was performed as described previously (7). Eyes were imaged using an AxioCam ERc 5s camera (Carl Zeiss) mounted on a Stemi 2000-C stereo microscope (Carl Zeiss).

#### Immunohistochemistry

*Drosophila* immunohistochemistry was performed as described previously (7). Primary antibodies used were: cleaved Dcp-1 (Cell Signaling Technology, 9578, 1:100), horseradish peroxidase-Cy3 (Jackson scientific, Stratech), Synaptotagmin (1:2000) (7), β-galactosidase (1:1000; MP Biologicals 0855976) and anti-elav (1:50, DSHB 9F8A9). All primary antibodies were incubated overnight at 4°C in PBS-T (0.1% Triton X-100), all secondary antibodies were incubated for 1h at room temp (∼21°C) in PBS-T. TUNEL staining was performed using TMR-red detection kit (Roche, 12 156 792 910).

#### Imaging and quantification

Quantification of synaptic bouton number at the *Drosophila* third instar larval neuromuscular junction (NMJ) was performed as described previously (7). Confocal microscopy was performed using a Zeiss LSM 880 on an Axio Observer.Z1 invert confocal microscope (Zeiss). Z-stacked projections of NMJ’s and VNCs were obtained using a Plan Neofluar 40×/0.75 NA oil objective. NMJ lengths were measured from stacked NMJ images using the NeuronJ plugin for ImageJ (National Institutes of Health) as described previously (7). Corrected total cell fluorescence (CTCF) quantification was performed as described previously using ImageJ (7). Neurons were identified in the *Drosophila* larval VNC using anti-elav.

#### Larval locomotor assay

Female third instar wandering larvae of the appropriate genotype were selected and transferred into HL3 (70 mM NaCl, 5 mM KCl, 1 mM CaCl_2_⋅2H_2_O, 10 mM NaHCO_3_, 5 mM trehalose, 115 mM sucrose and 5 mM BES in dH_2_O) to wash off any debris. Two to three larvae were transferred onto the center of a 90 mm diameter petri-dish containing a thin layer of 1% agar and left to acclimatize. The petri dish was placed upon a black surface and imaged from above using a digital webcam (Creative labs, UK). Experiments were performed at 25°C. Upon initiation of crawling larvae were recorded for 120 s (0.2 frames s^−^^1^) using VirtualDub software. Images were analyzed using imageJ. Briefly videos were batch thresholded and a custom macro used to track, via the MTrack2 plugin, and plot the larval positions. These data were then used to determine the mean larval velocity.

### Mouse

#### *Ex vivo* histology

Brains were isolated from 12-month aged CHMP2B^Wild-type^ and CHMP2B^Intron5^ expressing mice, described previously (30), and fixed in 4% formalin for 24 h. Tissue was embedded in paraffin blocks and 5 μm sagittal sections taken. Sections were deparaffinized by heating at 55°C for 10 min followed by further xylene deparaffinizaton and rehydrated in a graded series of ethanol. Heat mediated antigen retrieval was performed in sodium citrate buffer (10 mM sodium citrate, 0.05% Tween 20, pH 6.0, 95°C, 10 min) followed by Retrievagen (pH 6.0, 95°C, 10 min, BD Biosciences). Samples were blocked in 5% goat serum (TBST 0.025%, 1 h room temp.) followed by endogenous mouse IgG blocking [AffiniPure Fab Fragment Goat Anti-Mouse IgG (H + L), Jackson immune research, stratech]. Samples were incubated in primary antibodies [POSH (1:100, Proteintech, 14649-1-AP) and β3 tubulin (1:500, Sigma T-8660) 1% BSA, TBST 0.025%, overnight, 4°C] followed by secondary antibodies (goat anti-mouse FITC, goat anti-rabbit Cy3, 1 h, room temperature, Jackson scientific, stratech). Samples were mounted in Vectashield mounting media + DAPI (H-1200, Vector labs) and analyzed using a Zeiss LSM 880 confocal (Plan Neofluar 40×/0.75 NA oil objective). Relative abundance of POSH within mouse cortical neurons was determined via CTCF using ImageJ. Relative fluorescence intensity of POSH was normalized against relative fluorescence intensity of β3-tubulin. Three to five cortical neurons per animal, three animals per genotype were analyzed.

#### Cell culture

##### Animal groups

Timed mated female Wistar rats (Charles River UK) (RRID: RGD_737929) were maintained in accordance with the ARRIVE guidelines and the UK Animals (Scientific Procedures) Act (1986). Hippocampi were dissected from postnatal days 1 to 4 (P1–4) rat pups. Animals were euthanized using pentobarbital injection followed by cervical dislocation, according to Home Office guidelines. Hippocampal cell suspensions were obtained as previously described (Potter, 1989) and cultured in neurobasal medium (21103049, Thermo Scientific) supplemented with B27 (50×, 17504044, Thermo Scientific), glucose (35 mM final concentration, A2494001, Thermo Scientific), l-glutamine (1 mM, 25030032, Thermo Scientific), fetal calf serum (5%, Mycoplex, PAA), penicillin (100 U/ml) and steptomycin (100 µg/ml, 15140122, Thermo Scientific) and maintained at 37°C in 5% CO_2_.

Neurons were transfected at 12 days *in vitro* (DIV) with Lipofectamine 2000 (11668019, Thermo Scientific) with either FLAG-tagged *CHMP2B^wild-type^* or *CHMP2B^Intron5^*, described previously (13), and with POSH ShRNA 1 + 2 or Scrambled ShRNA’s (52; Supplementary Material, Fig. S1C). After 2/3 days, cells were fixed or lysed for biochemical experiments. GPNT’s (Sigma) were cultured in Ham’s F-10 (Lonza, BE12-618F) with l-glutamine supplemented with basic fibroblast growth factor (FGF, 2 ng/ml, Sigma F3685), heparin (80 µg/ml, Sigma H3149) and fetal calf serum (10%). Cells were plated in 35 mm dishes and at ∼70% confluency transfected with either 20 µg CHMP2B^Wild-type^ or CHMP2B^Intron5^. After 3 DIV, cells were lysed for western blotting. Culture media was routinely screened for mycoplasma contamination.

### Immunocytochemistry

Cells were washed with phosphate buffered saline (PBS) and fixed for 30 min at room temperature with 4% paraformaldehyde (containing 4% sucrose) (Sigma) PBS. Cells were permeabilized in 0.5% NP40 in PBS for 5 min at room temperature. Primary antibodies used were as follows: anti-FLAG (Sigma M2 clone, 1:1000), anti-GFP (eBioscience, 14-6758-81, 1:1000). dsRed was detected using FluoTag-X4, ATTO 542 (1:500, Synaptic Systems). Primary antibodies were incubated overnight at 4°C. Corresponding Alexafluor secondary antibodies (1:500, Thermo Scientific) were incubated for 1 h at room temperature before mounting with Fluoromount (Sigma).

#### Microscopy and image analysis

Images were collected on an inverted Zeiss microscope (880) with 20× or 63× Plan Neofluar objectives using Zeiss filter sets for DAPI and Alexa 488/546/633. Images were taken at an aspect ratio of 2048× 2048. Images of neurons were traced using the NeuronJ plugin in ImageJ (1.6.0). Individual traces were saved, thresholded and sholl analysis was conducted using the Sholl plugin.

### Biochemistry

#### Western blotting

Seven brains, per genotype, were dissected from third instar wandering larvae and boiled in 20 μl of 2× laemmli loading buffer. Cells were lysed in RIPA containing phosSTOP phosphatase inhibitors (Roche) and cOmplete EDTA free protease inhibitors (Roche). Cortical lysates were extracted from mouse brain as described previously (30). After boiling in loading buffer samples were run on a 4–20% Mini-PROTEAN^®^ TGX™ Precast Protein Gels (Biorad) prior to transfer onto standard PVDF membrane. Antibodies used for immunoblotting were Guinea Pig Anti-POSH (193, 1:5000), anti-Phospho-(Ser/Thr) Akt Substrate (Cell signaling Technology, 9611, 1:1000), anti-cleaved Dcp-1 (Cell Signaling Technology 9578, 1:1000), anti-β-Actin [Proteintech, 60008-1-Ig (7D2C10), 1:180 000], anti-Cleaved Caspase 3 (CC3) (1:200, Cell Signaling Technology 9661), anti-GAPDH [Merck, MAB374 (6C5), 1:10 000], anti-pJNK (Promega Active JNK, pTPpY, 1:5000) and anti-Pan-JNK (Cell Signaling Technology 9252, 1:1000). Secondary antibodies were peroxidase-conjugated AffiniPure Goat Anti-Guinea Pig, anti-rabbit and anti-mouse IgG (H + L) (Jackson Scientific, 106-035-003, 111-035-144 and 115-035-003, 1:10 000). For immunoblotting following immunoprecipitation secondary antibodies were peroxidase-conjugated protein-G (Merck Millipore 18–161, 1:10 000). POSH antibodies were produced from full length GST-tagged *Drosophila* POSH, generated from POSH cDNA (LD45365, Berkeley Drosophila Genome Project Gold Collection) and injected into Rabbit or Guinea Pig (eurogentec, 87-day immunization). Phospho mobility shift gels were performed by addition of Phos-tag acrylamide (AAL-107, Wako laboratory chemicals) to standard acrylamide gels as per the manufacturer’s instructions.

#### Immunoprecipitation

For immunoprecipitation protein was extracted from *Drosophila* in RIPA containing phosSTOP phosphatase inhibitors (Roche) and cOmplete EDTA free protease inhibitors (Roche). Lysates were incubated at a concentration of 1 mg/ml with Rabbit Anti-POSH (429, 4°C, overnight) followed by incubation with protein A-agarose beads (Sigma, P2545, 4°C, 4 h). Following incubation Protein A-agarose beads were collected using a Spin-X tube filter (Corning, Costar, 0.45 μm), washed and the sample eluted in 2× laemmli. Western blotting was performed as described above.

#### Statistics

Statistical analysis was performed using either SPSS Statistics (IBM, Version 24) or GraphPad Prism (6.01). Data are presented as mean values, from at least three biological replicates, with error bars representing the standard error of mean (SEM). Mean, SEM, statistical tests, *P*-values and sample sizes are reported in the figure legends.

## Supplementary Material

Supplementary Material is available at *HMG* online.

## Supplementary Material

Supplementary Figure S1
