# Supplementary figures and images for: The pro-apoptotic JNK scaffold POSH/SH3RF1 mediates CHMP2BIntron5-associated toxicity in animal models of frontotemporal dementia
Source: Hum Mol Genet. 2018 Feb 8;27(8):1382–95. doi: 10.1093/hmg/ddy048 (PMC6454437; doi:10.1093/hmg/ddy048)

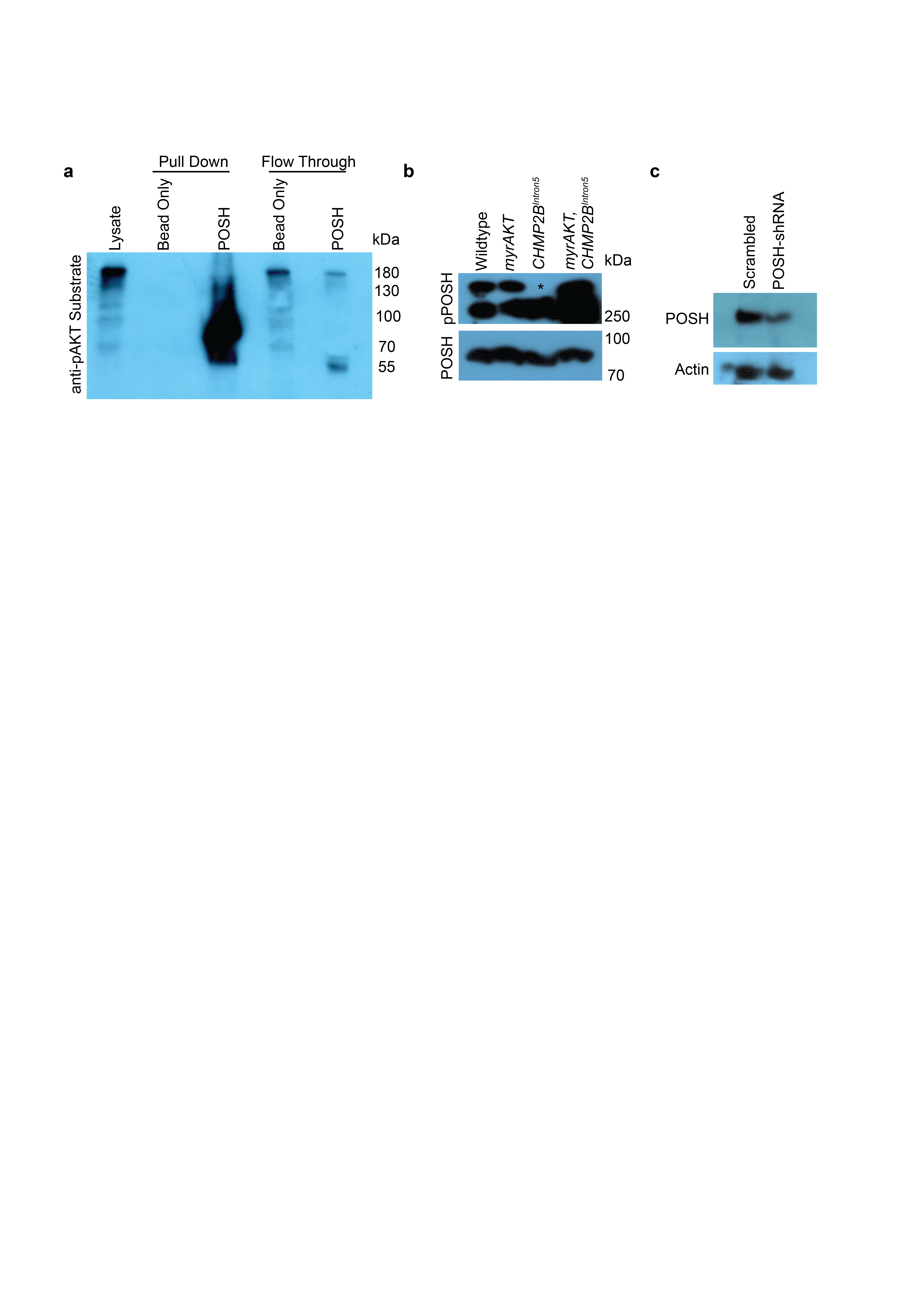

Supplement: Supplementary Figure S1 [file ddy048_fig_s1.png]
